# RACISM IN RESEARCH ON OLDER MIGRANTS AND ETHNO-RACIAL MINORITIES: INSIGHTS FROM TWO SCOPING REVIEWS

**DOI:** 10.1093/geroni/igad104.1315

**Published:** 2023-12-21

**Authors:** Sandra Torres

**Affiliations:** Uppsala University, Uppsala, Uppsala Lan, Sweden

## Abstract

The study of ethno-racialized minorities used to be primarily the domain of American social gerontologists. Meta-analyses of this scholarship have shown that this research has so far been characterized by two sets of practices: it engages in racialization but vaguely acknowledges racism, and it seems inequality-obsessed but justice-oblivious. Some gerontologists have therefore argued that the scholarly imagination that informs inquiries on older ethno-racialized minorities needs to be expanded, and that one way to do this could entail taking cues from European migration scholars’ inquiries on aging and old age. These scholars place the social position that is migrancy (rather than ethnicity and/or race) at the center of their scholarly imagination. This presentation presents the results of a scoping review of peer-reviewed literature published between 1990-2022 that focuses specifically on how the literature on older migrants engages with racism (since literature on older ethno-racialized minorities was in focus in another scoping review; 1998-2017). The review shows that although scholarship on older migrants recognizes that these axes of oppression could have an impact in their life-courses, empirical inquiries on the ways in which older people experience such oppression are still quite rare. The presentation will juxtapose the results of both scoping reviews in order to argue that our failure to inquire into older migrants’, and older ethno-racialized minorities’ experiences of racism impedes us from furthering our understanding of how the social positions that are ethnicity, race and migrancy impact (on their own and together) people’s experiences of aging and old age.

